# Tumor Spectrum, Tumor Latency and Tumor Incidence of the *Pten*-Deficient Mice

**DOI:** 10.1371/journal.pone.0001237

**Published:** 2007-11-28

**Authors:** Tsai-Ling Lu, Junn-Liang Chang, Chih-Chia Liang, Li-Ru You, Chun-Ming Chen

**Affiliations:** 1 Department of Life Sciences and Institute of Genome Sciences, National Yang-Ming University, Taipei, Taiwan; 2 Department of Pathology, Taoyuan Armed Forces General Hospital, Lungtan, Taoyuan County, Taiwan; 3 Institute of Biochemistry and Molecular Biology, National Yang-Ming University, Taipei, Taiwan; University of Hong Kong, China

## Abstract

**Background:**

*Pten* functionally acts as a tumor suppressor gene. Lately, tissue-specific ablation of *Pten* gene in mice has elucidated the role of *Pten* in different tumor progression models. However, a temporally controlled *Pten* loss in all adult tissues to examine susceptibility of various tissues to *Pten*-deficient tumorigenesis has not been addressed yet. Our goal was to explore the genesis of *Pten*-deficient malignancies in multiple tissue lineages of the adult mouse.

**Methods and Findings:**

We utilized an inducible Cre/*lox*P system to delete *Pten exon 5* in the systemic organs of *ROSA26 (R26)-CreER^T^;Pten^fx/fx^* mice. On reaching 45 weeks 4OHT-induced *Pten* loss, we found that the *R26-CreER^T^;Pten^fx/fx^* mice developed a variety of malignancies. Overall tumor mean latency was 17 weeks in the *Pten*-deficient mice. Interestingly, mutant females developed malignancies more quickly at 10∼11 weeks compared with a tumor latency of 21 weeks for mutant males. Lymphoma incidence (76.9% in females; 40.0% in males) was higher than the other malignancies found in the mutant mice. Mutant males developed prostate (20.0%), intestinal cancer (35.0%) and squamous cell carcinoma (10.0%), whereas the mutant females developed squamous cell carcinoma (15.4%) and endometrial cancer (46.1%) in addition to lymphomas. Furthermore, we tested the pharmacological inhibition of the PTEN downstream effectors using LY294002 on *Pten*-deficient prostate hyperplasia. Our data revealed that, indeed, the prostate hyperplasia resulting from the induced *Pten* loss was significantly suppressed by LY294002 (*p* = 0.007).

**Conclusions:**

Through monitoring a variety of *Pten*-deficient tumor formation, our results revealed that the lymphoid lineages and the epithelium of the prostate, endometrium, intestine and epidermis are highly susceptible to tumorigenesis after the *Pten* gene is excised. Therefore, this *R26-CreER^T^; Pten^fx/fx^* mouse model may provide an entry point for understanding the role of *Pten* in the tumorigenesis of different organs and extend the search for potential therapeutic approaches to prevent *Pten*-deficient malignancies.

## Introduction

Tumors occur in normal tissues that are challenged by a variety of genetic and environmental risk factors. These risk factors may consequently evoke aberrant effects on cytoplasmic signaling network, which result in uncontrolled cellular proliferation, cell survival and heterotypic cross-talk with neighboring cells. Among the various types of complicated cellular signaling circuitry that have been identified, the tumor suppressor *PTEN*, a phosphatase and tension homolog on chromosome 10, acts as the most important negative regulator of the phosphatidylinositol 3-kinase (PI-3K) signaling pathway [1], [2]. PTEN converts PIP3 to phosphatidylinositol (4,5) diphosphate (PIP2) in order to maintain a homeostasis of PIP3, which is generated by PI-3K activation [3]. Inactivation of PTEN results in an accumulation of PIP3, which recruits the serine/threonine kinase PKB/AKT to the cell membrane and this further activate downstream of the PI-3K/AKT signaling pathway to promote cellular proliferation, anti-apoptosis, cell survival and tumorigenesis [1], [4].

Mutation of *PTEN* is often seen in human cancers [5], [6], [7]. Using a gene targeting strategy to ablate *Pten* gene function in the mouse causes embryonic lethality between days 6.5 to 9.5 of gestation [8], [9], [10], [11]. The early lethality of *Pten*-deficient mice limits our understanding regarding how PTEN regulates downstream signaling and is related to tumorigenesis at adult stage. Up to this point, mice heterozygous for *Pten* have provided some basis for study because they develop a variety of cancers, including breast cancer, endometrial cancer, prostate tumors and lymphoma [8], [9], [11], [12]. Loss-of-heterozygosity of *Pten* might contribute to the tumor development in *Pten^+/−^* mice[9], [11], [12]. In addition, other genetic lesions such as *Nkx3.1*, *Trp53* or *p27KIP1* might be involved in decreasing latency and increasing invasiveness/metastasis during tumor development in *Pten^+/−^* mice [13], [14], [15], [16], [17].

Using Cre-loxP conditional genetics [18], [19], tissue-specific inactivation of *Pten* results in tumor formation in the targeted tissues of the mouse. Prostate-specific ablation of *lox*P-flanked *Pten* gene (*Pten^fx/fx^*) utilizing different Cre transgenic mice lines such as probasin-Cre, prostate-specific antigen (PSA)-Cre or mouse mammary tumor virus (MMTV)-Cre has shown the occurrence of prostate cancer progression similar to that which occurs in humans [20], [21], [22], [23]. Likewise, conditionally ablating the *Pten* gene in hematopoietic lineages causes various leukemias in the mouse [24]. Such Cre activity might also be present in the disease-initiating stem cells from which the *Pten* gene is deleted, which is followed by tumor initiation, expansion and progression [24], [25], [26]. Thus, tissue-specific and cell type-specific *Pten* knockout mice have provided a fundamental basis for an understanding of the role of *Pten* in different tumor progression models. However, somatic inactivation of *Pten* in a temporally controlled manner in all adult tissues to test susceptibility of various tissues to *Pten*-deficient tumorigenesis has not been carried out up to the present.

To assess the tumor incidence, latency and spectrum of *Pten*-deficient adult mice, we utilize two mouse strains: firstly, the *ROSA26 Cre-estrogen receptor (R26-CreER^T^)* knock-in mouse line, which expresses inducible Cre recombinase driven by the ubiquitous promoter *ROSA26* [27] and secondly, the *Pten^fx/fx^* mouse carrying *lox*P-flanked exon 5 encoding the phosphatase domain of PTEN [28], [29]. 4-hydroxytamoxifen (4-OHT) is administrated to induce the *Pten* gene excision in a temporally controlled manner in the crossed mutant offspring (*R26-CreER^T^/+;Pten^fx/fx^*). In this report, we found that lymphoid lineages, the squamous epithelium of the epidermis, the simple epithelium of the prostate, the endometrium and the intestine are highly susceptible to tumorigenesis in such a *Pten*-deficient background. Application of the PI3K inhibitor LY294002 in these *Pten*-deficient mice resulted in the enlargement of the anterior prostate glands being significantly suppressed. Thus, we have established a *Pten*-deficient tumor model, which demonstrates that multiple cell lineages can be provoked to undergo aberrant cellular signaling simultaneously and that this results in major changes of normal tissues.

## Results

### Temporally controlled CreER-mediated recombination by 4OHT administration

**Figure 1:**
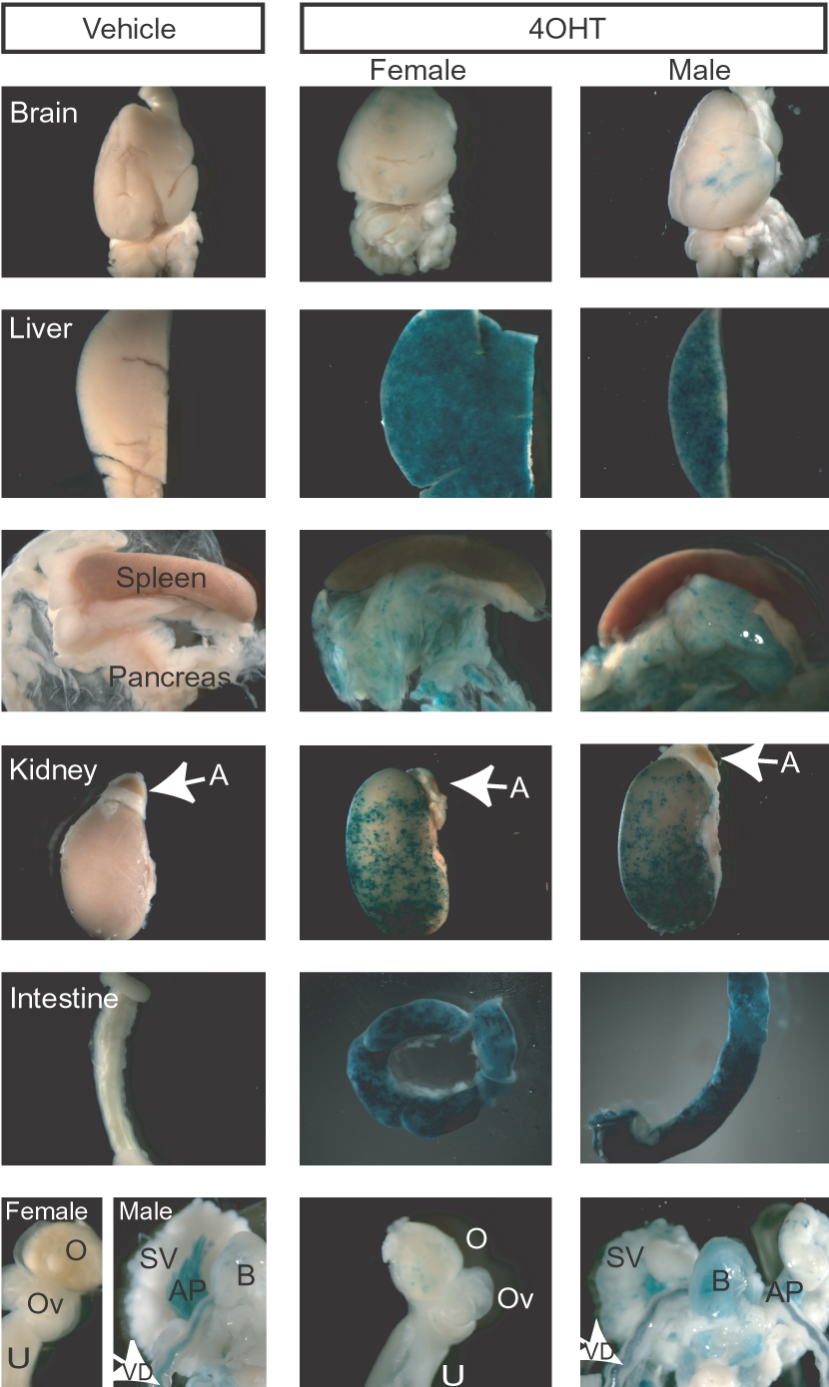
Evaluation of 4OHT-induced Cre recombination in *R26CreER^T^; R26R* mice. After 10 days of 4OHT or vehicle control (DMSO) injection, *LacZ* reporter activity in the systemic organs including brain, liver, spleen, pancreas, kidney, intestine and reproductive tracts of *R26CreER^T^;R26R* mice (2 males and 2 females) were examined by X-gal staining and compared with those of the control mice. The representative X-gal staining patterns in the systemic organs of a DMSO-treated control male mouse and the reproductive organs of a control female mouse are shown in the left panels. Representative X-gal positive (blue) patterns reflecting inducible Cre activity are shown in the other panels (middle, 4OHT-treated *R26CreER^T^;R26R* female; right, 4OHT-treated *R26CreER^T^;R26R* male). A, adrenal gland; AP, anterior prostate; B, bladder; O, ovary; Ov, oviduct; SV, seminal vesicle; U, uterus; VD, vas deferens

The inducible Cre activity of the *R26-CreER^T^* transgenic mice was demonstrated in neuronal tissues by Badea et al. [27]. In this report, we have examined temporally controlled Cre activity in the systemic organs of *R26-CreER^T^; R26R* bigenic mice. After 4OHT treatment for one week, whole mount X-gal staining revealed that the 4OHT-induced β-galactosidase expression showed focal or mosaic blue patterns in the brain, liver, pancreas, kidney, intestine, uterus and bladder of *R26-CreER^T^;R26R* bigenic males or females (**Figure 1**). Non-specific X-gal staining was also observed in the hind-stomach, the gut, the prostate and the vas deferens (**Figure 1** and data not shown). These results suggested that the inducible Cre activity of *R26-CreER^T^* transgenic mice had been successfully controlled by 4OHT in a variety of organs, although the level of inducible Cre activity may have differed as indicated by the variable X-gal stained intensity among these systemic organs.

**Figure 2:**
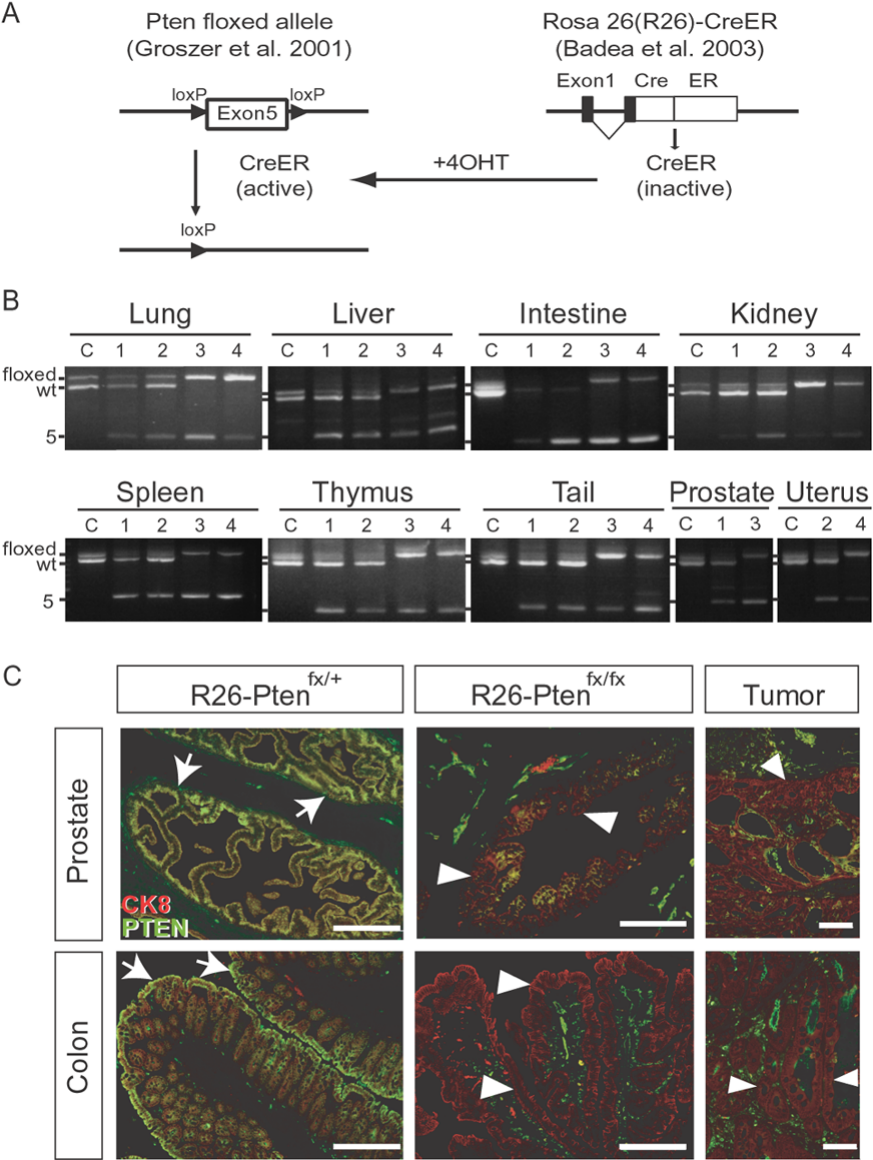
Temporally controlled *Pten* loss in *R26-CreER^T^/+;Pten^fx/fx^* mice. (A) Illustration of *Pten* conditional (floxed; fx) allele, *ROSA26-CreER^T^* transgene and 4OHT-induced *Pten exon 5* excision. The *CreER^T^* fusion protein (inactive) was initially expressed under a ubiquitous promoter ROSA26. The 4OHT inducer was used to temporally active the CreER^T^ recombinase activity, which resulted in the excision of the DNA fragment containing the *Pten exon 5.* (B). PCR genotyping of the Pten floxed allele and Cre-mediated exon 5 excision; After one week of 4OHT injection, genomic DNA was isolated individually from multiple organs including the lung, the liver, the intestine, the kidney, the spleen, the thymus, the tail, the anterior prostate and the uterus of mice carrying different genotypes (C, *Pten^fx/+^*; 1, *R26-Pten^fx/+^* male; 2, *R26-Pten^fx/+^* female; 3, *R26-Pten^fx/fx^* male; 4, *R26-Pten^fx/fx^* female). PCR genotyping is shown that allows the identification of the Pten floxed allele (∼1100-bp), the wild-type (∼1000-bp) and the exon 5 excised alleles (Δ5; ∼400-bp) from the 4OHT-treated mice. (C). Immunostaining of PTEN in the prostate and the colon. Representative merged images of the immunofluorescence analysis reveal the expression level of PTEN in the anterior prostate lobe (at 7 weeks post-4OHT treatment) and the colon (at 37 weeks post-4OHT treatment) of the *R26-Pten^fx/+^* and *R26-Pten^fx/fx^* mice. Antibody against PTEN (green) and TROMA-I antibody against cytokeratin 8 (CK8; red) were used to study the expression of PTEN in the simple epithelial subsets. Arrows indicated the coexpression of PTEN and CK8 (yellow) in anterior prostate gland and colon epithelium of *R26-Pten^fx/+^* mice (left panels), whereas the hyperplastic epithelial subsets (middle panels; red) of the *R26-Pten^fx/fx^* mice showing PTEN loss (arrowheads). In addition, the immunofluorescence images in the right panels reveal PTEN loss in the tumor lesions of prostate and colon of *R26-Pten^fx/fx^* males (at 13 and 39 weeks, respectively). Scale Bar, 200 µm.

To investigate tumor susceptibility in the *Pten*-deficient mice at an adult stage, we generated *R26-CreER/+;Pten^fx/fx^* (referred to as *R26-Pten^fx/fx^* hereafter) and control (*R26-Pten^fx/+^*, *Pten^fx/+^* or *Pten^fx/fx^*) mice and followed this with 4OHT induced Cre/loxP recombination to excise exon 5 of *Pten* gene (**Figure 2A**). After 4OHT treatment for one week, we examined the efficiency of exon 5 of *Pten* gene excision using the genomic PCR method in a variety of organs dissected from males and females carrying the *Pten^fx/+^*, *R26-Pten^fx/+^* and *R26-Pten^fx/fx^* genotypes (**Figure 2B**). Our result showed that exon 5 of the *Pten* gene excision was detected in *R26-Pten^fx/+^* and *R26-Pten^fx/fx^* tissues, indicating that 4OHT was able to induce Cre-mediated excision of *lox*P-flanked region in the tissues examined (**Figure 2B**). Notably, the excision efficiency of *Pten* showed no overt differences between males and females carrying the *R26-Pten^fx/+^* or *R26-Pten^fx/fx^* genotypes. However, inducible Cre activity may vary slightly across the organs of the *R26-Pten^fx/+^* or *R26-Pten^fx/fx^* mice (**Figure 2B**). We next examined the expression of PTEN protein in selected organs, specifically the anterior prostate and the intestinal tract, of 4OHT-injected *R26-Pten^fx/fx^* and control (*R26-Pten^fx/+^*) mice using immunofluorescence microscopy. Our results showed that PTEN could be detected in the prostate and colon epithelium, which was indicated by cytokeratin 8 (CK8) staining of the controls (yellow; CK8-positive, PTEN-positive). In contrast, PTEN expression was lost in most of the hyperplastic epithelial lesions and tumor lesions (red; CK8-positive, PTEN-negative) found in the corresponding organs of the *R26-Pten^fx/fx^* mice (**Figure 2C**). We also examined the expression of PTEN in various other systemic organs, namely the lung and the kidney, of *R26-Pten^fx/fx^* and the control mice to demonstrate PTEN loss (**Figure S1**). Our results suggested that the majority of PTEN was lost from the systemic organs of the *R26-Pten^fx/fx^* mice (**Figure 2B & C**; **Figure S1**).

### Analysis of *R26-Pten^fx/fx^* and *R26-Pten^fx/+^* malignancies

**Figure 3:**
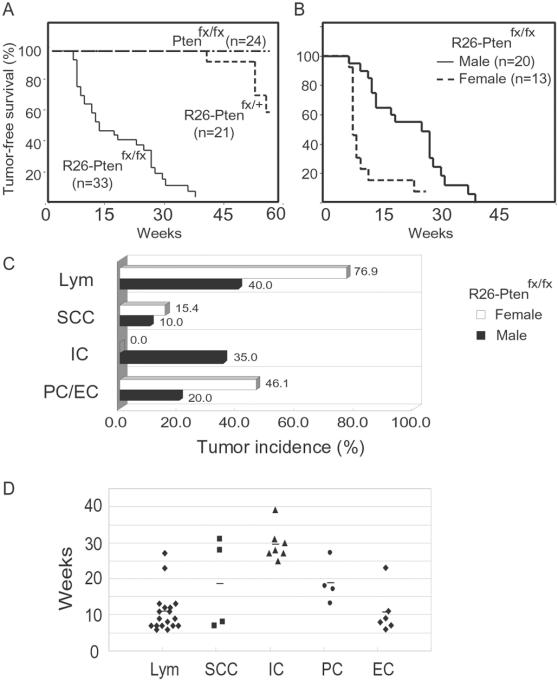
Tumor-free survival, tumor spectrum and tumor incidence of *R26-Pten^fx/fx^* mice. (A) Tumor-free survival of *Pten^fx/fx^*, *R26-Pten^fx/+^* and *R26-Pten^fx/fx^* mice; (B) Tumor-free survival of male and female *R26-Pten^fx/fx^* mice; (C) Tumor spectrum and incidence of male and female *R26-Pten^fx/fx^* mice; Lym, lymphomas; PC, prostate cancer; EC, endometrial cancer; IC, intestinal cancer; SCC, squamous cell carcinoma; The percentage of each tumor type is shown on the right of bar graph. (D) Tumor latency of the *R26-Pten^fx/fx^* mice; each dot represents an individual malignant tumor in the *R26-Pten^fx/fx^* mice. ⧫, Lymphoma (Lym); ▪, Squamous cell carcinoma (SCC); ▴, Intestinal cancer (IC); •, prostate cancer (PC); Bars represent the mean tumor latency.

Furthermore, *R26-Pten^fx/fx^* (n = 33; 20 males and 13 females) and control (*Pten^fx/fx^*, n = 24; *R26-Pten^fx/+^*, n = 21) mice were given 4-OHT injection (i.p.) at age of 6 weeks and then monitored for tumor development over about 60 weeks. The mice were sacrificed for pathological analysis (H&E and IHC) when they manifested sign of distress or on tumor detection. The cumulated tumor-free survival curves of the *R26-Pten^fx/fx^* and control mice are shown in **Figure 3A**. Our results showed that all the *R26-Pten^fx/fx^* mice died from their tumor burden by 45 weeks post 4OHT injection (**Table 1**). The overall mean latency of *Pten*-deficient tumor formation was 17 weeks. In addition, we found 4 *R26-Pten^fx/+^* mice (19.0%) developed various different types of tumors by 60 weeks post 4OHT treatment (**Table 1**). We found that one *R26-Pten^fx/+^* mouse suffered from metastatic lung cancer at 42 weeks, two *R26-Pten^fx/+^* females developed mammary tumors at 52 and 55 weeks and another *R26-Pten^fx/+^* male mouse developed hepatocellular carcinoma at 58 weeks (**Table 1**; **Figure S2**). No tumors were found in the control group *Pten^fx/fx^* mice at 60 weeks.

**Table 1:** Malignancies of *R26-Pten^fx/+^* and *R26-Pten^fx/fx^* mice

	*R26-Pten^fx/+^*	*R26-Pten^fx/fx^*
Total number	21	33
Male	16	20
Female	5	13
Tumor incidence (%)	4 (19.1)	33 (100.0)
Tumor spectrum (%)		
Lymphoma	0	18 (46.2)
Lung cancer	1 (25.0)	0
Liver cancer	1 (25.0)	0
Intestinal cancer	0	7 (17.9)
Prostate cancer	0	4 (10.3)
Endometrial cancer	0	6 (15.4)
Breast cancer	2 (50.0)	0
Squamous cell carcinoma	0	4 (10.3)
Total tumor number	4	39*

* Multiple tumors were found in *R26-Pten^fx/fx^* mice (5 females and 4 males).

Interestingly, we found that the tumor latency and spectrum of the *Pten*-deficient mice exhibited gender differences. Approximately, 50% *R26-Pten^fx/fx^* males and females had malignant tumors at 21 weeks and between 10 and 11 weeks, respectively (**Figure 3B**). Most *R26-Pten^fx/fx^* females quickly developed lymphomas (10 of 13; 76.9%) and died at about 9 weeks post-4OHT injection compared to a much lower incidence of lymphoma formation (8 of 20; 40%) by 13 weeks in the males (**Figure 3C**). The *Pten*-deficient male mice developed intestinal cancers (7 of 20, 35%) arising from the colorectum (n = 6) and small intestine (n = 1), whereas no female mice developed this type of cancer. This might be partly due to the quick development of the lymphomas, which caused early lethality before the intestinal cancer could develop in the *R26-Pten^fx/fx^* females. In addition, the *R26-Pten^fx/fx^* mice developed squamous cell carcinoma of the epidermis (4 of 33 mice, 12.1%; 2 males, 10% and 2 females, 15.4%), endometrial cancer (6 of 13 females, 46.1%) and prostate cancer (4 of 20 males, 20%) (**Figure 3C**). The mean latency of these malignancies is showed in **Figure 3D**. The sequential occurrence of the *Pten*-deficient malignancies was about 11 weeks for the lymphomas and the endometrial cancer, about 19 weeks for the prostate cancer and squamous cell carcinoma and about 30 weeks for the intestinal cancer (**Figure 3D**).

**Figure 4:**
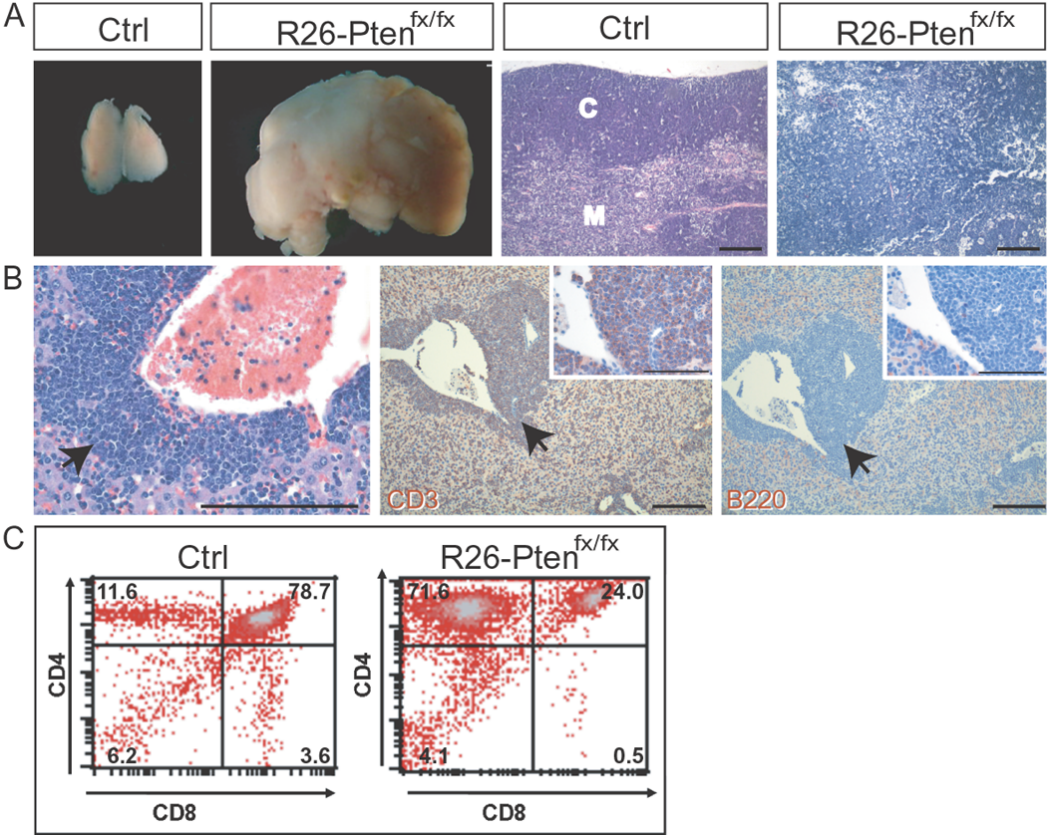
Characterization of *R26-Pten^fx/fx^* lymphomas. (A) Gross morphology of a *R26-Pten^fx/fx^* thymic tumor enlarged compared to the control (ctrl) thymus. H&E stained section of a *R26-Pten^ fx/fx^* thymic tumor reveals the morphology of the small lymphocytic cells in comparison to the control (ctrl). C, cortex; M, medulla; Scale Bar, 200 µm (B) Infiltrated lymphocytic tumor cells (arrows) in the liver of a *R26-Pten^fx/fx^* mouse characterized by H&E (left) and IHC antibodies against CD3 (middle; positive stained brown cells) and B220 (right; negative stained blue cells), which allow the assessment of the origin of the tumor. Scale bar, 200m; High-power views of CD3 or B220 IHC are inserted (scale bar, 100 µm). (C) Flow cytometry analysis of thymocyte suspensions from a control and a *R26-Pten^fx/fx^* mouse stained with antibodies against CD4 and CD8. Percentage of cells is indicated in each quadrant.

The lymphomas mainly arose from the thymus and mesenteric lymph nodes. A representative thymic lymphoma is shown grossly in **Figure 4A**. H&E staining revealed a mature appearance for the small lymphocytic cells and a loss of cortical-medullary architecture in this thymic tumor (**Figure 4A**). To verify the potential origins of this lymphoma, T-cell and B-cell origins were respectively determined by antibodies against CD3 and B220 in the liver with the infiltrated lymphoid tumor cells in the portal area identified using IHC, which showed that this tumor was a CD3-positive T-cell lymphoma (**Figure 4B**). Through evaluation of CD3 and B220 expression in the lymphomas formed in the mutant mice, we found that all lymphomas were derived from CD3-positive T-cells with either a mature appearance of small lymphocytes or large lymphoblasts (data not shown). Occasionally, the more detail surface markers that were expressed on these lymphomas were analyzed by flow cytometry in order to assist our verification of these lymphomas. As an example, a CD4-rich T-cell lymphomas was found to have developed in a *R26-Pten^fx/fx^* mouse at six weeks post-treatment (**Figure 4C**). We also found that one mouse developed both CD4- and CD8-rich lymphomas arising from the thymus and mesenteric lymph node, respectively, suggesting that 4OHT-induced *Pten* loss in different lymphoid origins may develop simultaneously (data not shown).

**Figure 5:**
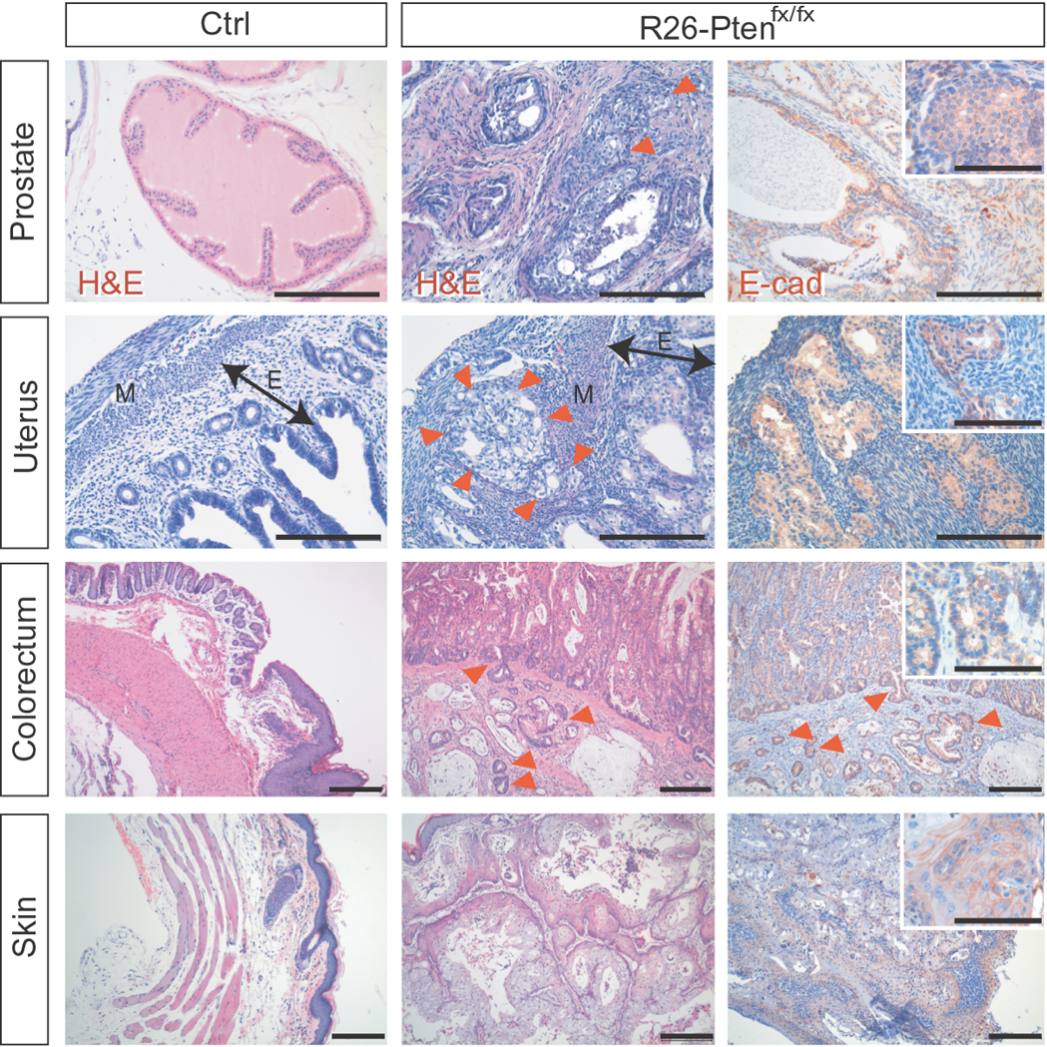
* R26-Pten^fx/fx^* epithelial malignancies. Representative histology (middle panels; H&E) and E-cadherin IHC (right panels; E-cad; NovaRed) of malignant tumors of *R26-Pten^fx/fx^* prostate, uterus, colorectum and skin tissue are showed to compare with the normal corresponding organs (left panels; H&E) from control mice. Red arrowheads indicated the invasive border of the prostatic, endometrial and colorectal cancers. High-magnification views of E-cadherin IHC revealing intense membranous staining are shown in the inserted photos (Bars, 100 µm). Bidirectional arrows indicated the endometrial portions. M, myometrium; E, endometrium; Scale bars, 200 µm.

**Figure 6:**
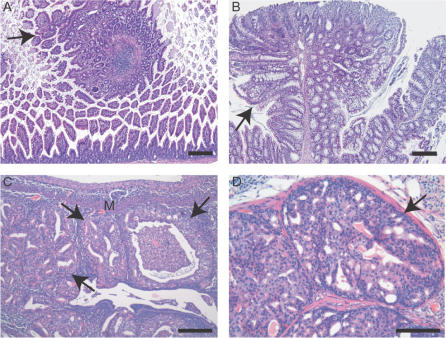
Pre-cancer lesions of *R26-Pten^fx/fx^* mice. (A & B) Representative H&E stained sections revealed the polyps of the small intestine (A) and the colon (B) of *R26-Pten^fx/fx^* males at 31 and 39 weeks 4OHT post-treatment, respectively. (C) Representative H&E stain revealing complex atypical hyperplasia of the endometrim was found in a *R26-Pten^fx/fx^* female, which also suffered from lymphoma, at 11 weeks 4OHT post-treatment. M, myometrium; (D) Representative histology of high grade PIN was found in a *R26-Pten^fx/fx^* male at 7 weeks post 4OHT injection. Arrowheads indicated the dysplastic epithelial lesions of the intestine (A & B), the uterus (C) and the anterior prostate gland (D) of *R26-Pten^fx/fx^* mice. Scale bars, 200 µm.

In addition to lymphomas, prostate cancer, endometrial cancer, intestinal cancer and squamous cell carcinoma of the epidermis were found in *R26-Pten^fx/fx^* mice using H&E and IHC analyses (**Figures 3** **&** **5**). The focal invasions of the prostate, endometrial and colorectal carcinomas were observed and characterized by H&E or IHC using antibodies against E-cadherin (**Figure 5**). Pre-cancer lesions (hyperplasia/dysplasia and adenoma) of corresponding malignancies were also observed in the *R26-Pten^fx/fx^* mice (**Figure 6**). We found that *R26-Pten^fx/fx^* males had developed small or large intestinal polyps at late onset (>35 weeks; **Figure 6A & B**). In addition, most mutant females developed endometrial hyperplasia (7 of 13) at between 7 and 9 weeks post-4-OHT injection (**Figure 6C**). The early alternations associated with prostate hyperplasia (BPH) or prostate intraepithelial neoplasm (PIN) were observed at 4∼6 weeks post 4-OHT injection (**Figure 6D**).

Moreover, multiple tumors were also observed in *R26-Pten^fx/fx^* mice (9 of 33, 27.3%; 5 females and 4 males). Among these animals, all females developed T-cell lymphoma and endometrial cancer at 7∼23 weeks post-4OHT injection. One of these females also developed squamous cell carcinoma additionally. Furthermore, two males developed prostate cancer together with lymphoma (at 13 weeks) and colorectal cancer (at 27 weeks), respectively. Finally, two males developed squamous cell carcinomas and colorectal cancer at 28 and 31 weeks post-4OHT injection.

### Temporally controlled prostate tumorigenesis

**Figure 7:**
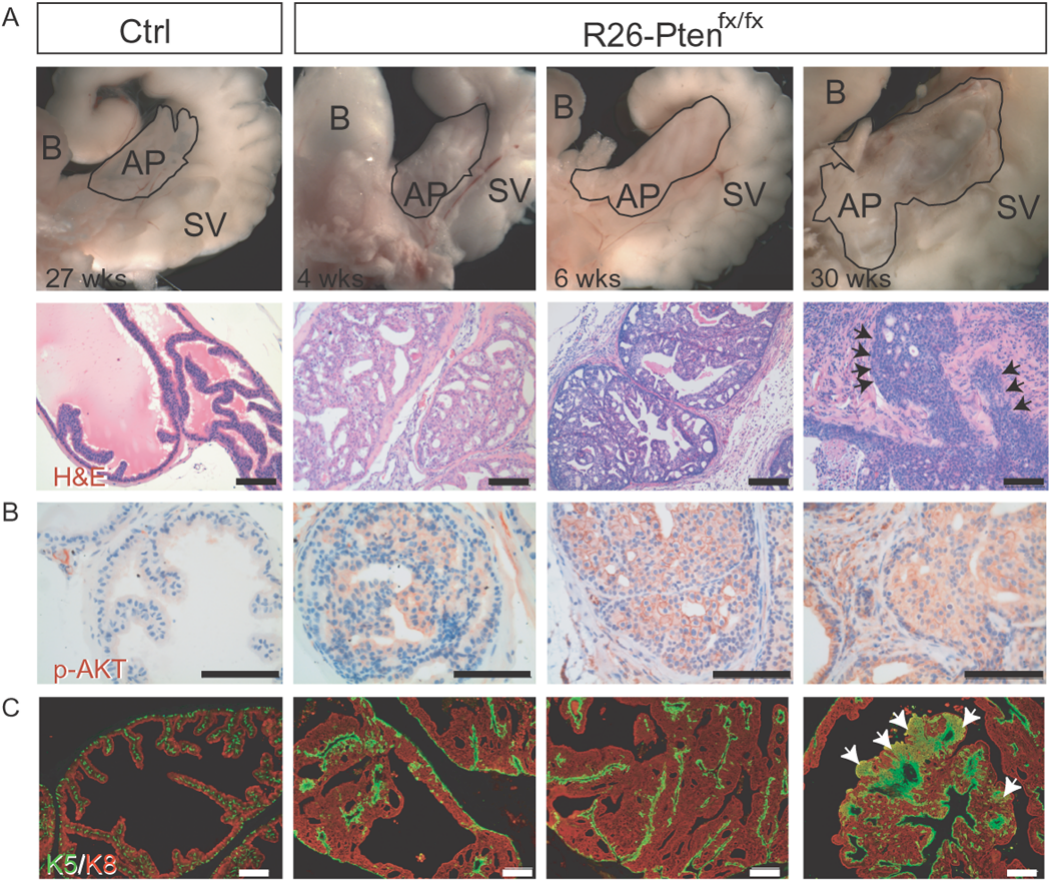
Prostate tumor progression of the *R26-Pten^fx/fx^* prostate. (A) Gross view (upper panels) and H&E staining (lower panels) of the anterior prostate of control (at 27 weeks 4OHT post-treatment) and of *R26-Pten^fx/fx^* mice (at 4 weeks, 6 weeks and 30 weeks after 4OHT post-treatment); B, bladder; SV, seminal vesicle; AP (encircled in upper panels), anterior prostate; Arrows indicate the invasive border of the prostate epithelium and stroma. (B) IHC of p-AKT (Ser473) reveal the strong membranous localization of p-AKT in the BPH, PIN and prostate cancer samples of the *R26-Pten^fx/fx^* mice compared to the normal epithelium of the control mice. (C) IF with antibodies against CK5 (green) and CK8 (red) reveal stratification and expansion of the basal and lumen epithelium in the BPH, PIN and prostate cancer samples of the *R26-Pten^fx/fx^* mice compared to the normal epithelium of the control mice. Arrows indicate the focal CK5/CK8 (K5/K8) double-positive cells.

The advantage of the use of inducible CreER^T^/*lox*P technology in this report is that it allowed the monitoring of tumor development at specific time points after 4-OHT injection. To investigate the progression of *Pten*-deficient malignancies, prostate cancer progression was chosen as the initial model because a good understanding of the role of *Pten* in this disease has been gained from other prostate-specific *Pten* knockout mouse model studies [20], [21], [23], [25]. At 4 weeks, 6 weeks and 30 weeks after 4-OHT injection, we found gradually enlargement of the anterior prostate in *R26-Pten^fx/fx^* mice compared to the controls (**Figure 7A**). Histologically, these prostate lesions were studied at the above time points (4, 6 and 30 weeks) and this revealed the prostate cancer progression through BPH/PIN to invasive cancer (**Figure 7A**). We then determined activation of AKT using antibody against phosphorylated AKT (p-AKT-Ser473) specifically. We found that AKT phosphorylation appeared in all prostate lesions along with hyperplasia and neoplasia in the *R26-Pten^fx/fx^* mice, unlike the controls (**Figure 7B**). To identify the potential cell type of the *Pten*-deficient prostate cancer, we performed double immunofluorescent staining to verify the presence of cytokeratin 5 (CK5^+^)-expressing basal cells and cytokeratin 8 (CK8^+^)-expressing lumen epithelial cells from the normal, BPH, PIN and prostate cancer samples in the *R26-Pten^fx/fx^* mice compared with the controls. Flattened and discontinuous CK5^+^-expressing cells were observed underneath the CK8^+^-expressing lumen epithelium in the control prostate, whereas stratification of the CK8^+^ lumen epithelium together with flatten CK5^+^ basal cells were noticed in the BPH/PIN lesions (**Figure 7C**). As the disease progressed to invasive prostate cancer in the *R26-Pten^fx/fx^* males, focal expansion of the CK5^+^ cells and coexpression of the CK5^+^/CK8^+^ cells were significantly increased (**Figure 7C**). However, the significance of the increased CK5/CK8 double positive cells in the *Pten*-deficient prostate cancer remains unclear.

### The PI3K inhibitor, LY294002, suppresses prostate hyperplasia

**Figure 8:**
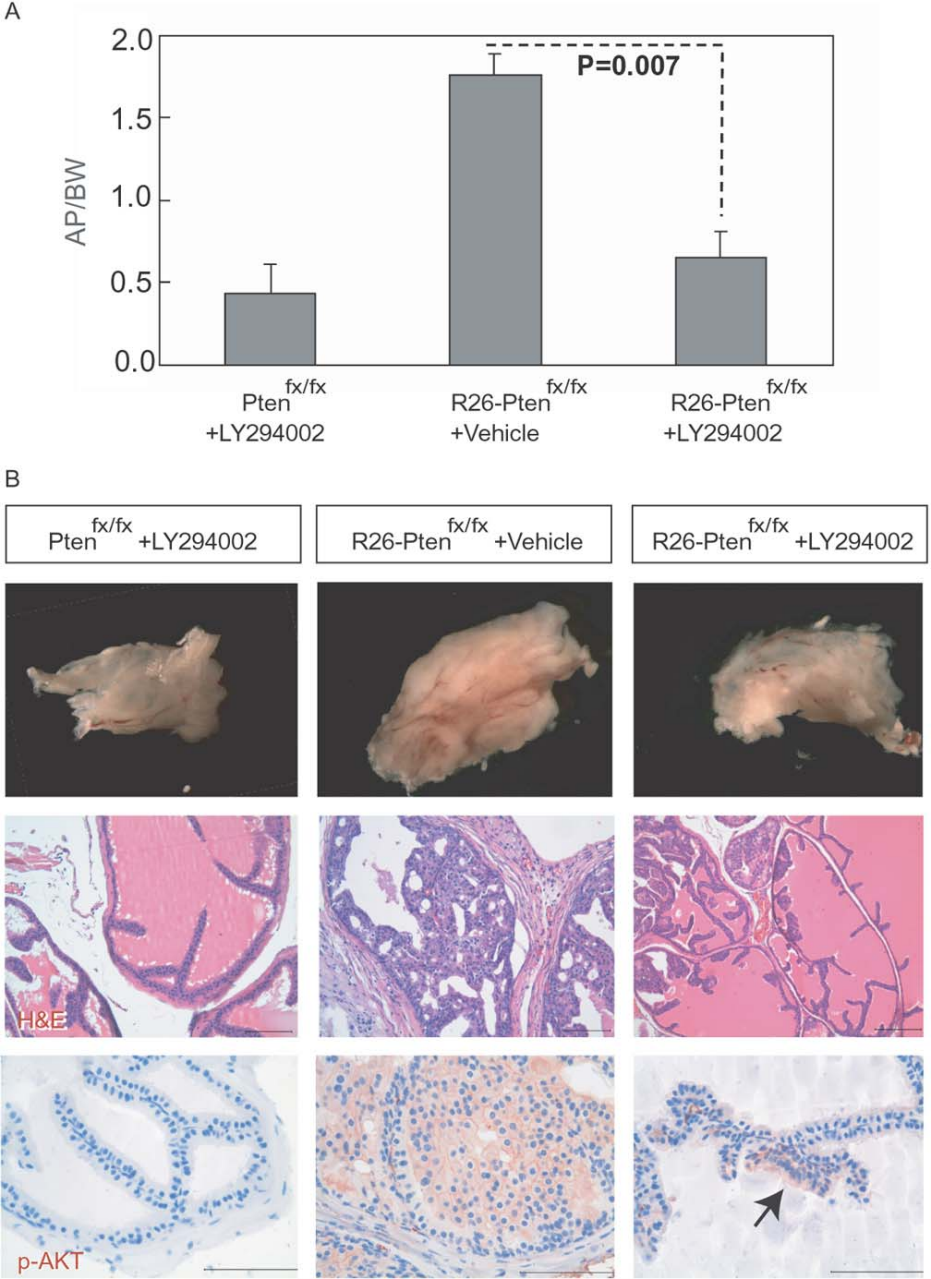
Suppressive effect of LY294002 on the *Pten*-deficient prostate enlargement. (A) The bar graph represented the relative weight (mean) of the anterior prostate lobe (AP weight in mg/body weight in g) of each group (n> = 3). The *p* value was calculated by Student's *t*-test. Error bars represent the standard derivation. (B) Dissected anterior prostates of control (*Pten^fx/fx^*) and of *R26-Pten^fx/fx^* mice in the absence or presence of LY294002 for 4 weeks; these are shown as a gross view (upper panels) and with H&E staining (middle panels). IHC of p-AKT(Ser473) is shown in the lower panels. Arrow indicates the focal positive for p-AKT in the LY294002-treated *R26-Pten^fx/fx^* prostate.

We next evaluated the effect of the potential cancer targeting agent LY294002, which is a potent inhibitor of PI3K-AKT signaling pathway in *Pten*-deficient prostate tumor progression. After LY294002 treatment for 4 weeks, we found that the apparent increased anterior prostate weight in *R26-Pten^fx/fx^* mice (n = 4) was significantly suppressed (*p* = 0.007) in comparison to the controls (LY294002-treated *Pten^fx/fx^* mice, n = 3; vehicle-treated *R26-Pten^fx/fx^* mice, n = 3; **Figure 8A**). Histological H&E staining revealed that the increased cellularity and nuclear atypia of the anterior prostate were diminished in LY294002 treated *R26-Pten^fx/fx^* mice compared with the untreated mice (**Figure 8B**). We next examined whether LY294002 treatment blocked the PI3K-AKT signaling pathway. Using IHC, we found that only focal epithelial lesions showed pAKT(Ser473)-positive staining in LY294002 treated *R26-Pten^fx/fx^* prostates, suggesting that LY294002 treatment strongly suppresses AKT phosphorylation at Ser473 in *R26-Pten^fx/fx^* mice compared to the untreated animals (**Figure 8B**). Therefore, our results demonstrated that inhibition of the PI3K-AKT pathway was sufficient to suppress *Pten*-deficient prostate hyperplasia.

## Discussion

In this study, we demonstrated that the tumor spectrum, tumor incidence and tumor latency during temporally controlled *Pten* loss in the mouse. This is the first *in vivo* analysis of *Pten*-deficient tumorigenesis in the systemic organs of the adult mouse. It is likely that lymphoid lineages and the epithelium of the prostate, endometrium, intestine and epidermis are highly susceptible to tumorigenesis after the *Pten* gene is excised by 4OHT-induced Cre/*lox*P recombination in the mouse's adult tissues. Although we have observed that Cre-mediated *Pten* excision did slightly vary among the systemic organs of *R26-Pten^fx/+^* or *R26-Pten^fx/fx^* mice, the variation seemed to show no correlation with the occurrence of *Pten*-deficient malignancies. Neoplastic cells may initiate from a single cell or a small number of cells because they are highly susceptible to tumorigenesis after PTEN loss and therefore slight variations in the Cre-mediated *Pten* excision will not affect the presence of such foci. Clinical studies have shown that somatic alterations of *PTEN* are found in proximately 5% of lymphomas[30], 29% of prostate cancer [31] and 34% of endometrial cancer [32]. Moreover, germline *PTEN* mutation shows strong linkage to Cowden syndrome (CS) and Bannayan-Zonana syndrome (BZS). These are inherited tumor predisposition syndromes in which hamartomas may develop in the central nervous system, gastrointestinal tract, skin, breast and thyroid [33]. Affected individuals of CD families also have a higher risk of malignancy in corresponding tissues [33], [34], [35]. Thus, our findings for the *R26-Pten^fx/fx^* tumor malignancies resemble to some degree of the tumor spectrum observed in human *PTEN* genetic studies.

Interestingly, the tumor latency and tumor spectrum of *R26-Pten^fx/fx^* mice exhibit gender-bias. *Pten*-deficient females are predisposed to lymphoma with an early onset of about 7 weeks, which may preclude the formation of other malignancies; the result of this may be the overall gender differences in the tumor spectrum. Particularly, intestinal cancer was not observed in *Pten*-deficient females, which may partly be due to the longer period time required to develop intestinal cancer, namely 25 weeks to 35 weeks post 4OHT treatment in mutant male mice.

Furthermore, we observed that only four mice heterozygous for the *Pten* floxed allele, among 21 *R26-Pten^fx/+^* mice analyzed, developed a variety of malignancies (up to 60 weeks).The incidence (19.0%, 4 of 21), latency (42 to 58 weeks) and spectrum (lung, breast and liver cancers) of the malignant tumors in our *R26-Pten^fx/+^* mice is quite different from that of a previous reported *Pten^+/−^* mouse model [9]. This report described approximately 18% lymphomas, 22% uterine cancer and 49% breast cancer occurring at ages ranging from 26 to 65 weeks. This discrepancy may be explained by the focal or mosaic excised activity of Cre/*lox*P-mediated recombination in the *R26-Pten^fx/+^* mice. In addition, it is possible that the genetic background of the two mouse models or some other undefined mechanism may affect the tumor latency and spectrum between the different mouse models of *Pten* heterozygosity [8], [11], [12], [36]. In a previous study, Freeman et al. reported that tumorigenesis in *Pten* heterozygotes was strongly affected by the genetic background of the mouse [36]. Mutant mice with a 129;C57BL6 background tend to have a higher incidence of lymphoid and endometrial malignancies. In contrast, *Pten* heterozygotes with a 129;BALB/c background develop prostate cancer more frequently. These findings are significant because they suggest that there are different modifier(s) present in different mouse strains that affect tumorigenesis in the mice after *Pten* loss. However, it remains unclear currently whether such background differences or putative modifier(s) are able to efficiently contribute to tumorigenesis in bi-allelic *Pten* loss. This might be further examined by generating *R26-Pten^fx/fx^* mice with different genetic backgrounds or different mutant strains in the future.

Although the multiple tumors that occurred simultaneously in the *Pten*-deficient mice may have complicated the monitoring of tumor formation compared to other mouse models that have used *Pten* ablation in a tissue-specific manner, our study is able to provide knowledge that will help an understanding of *Pten* tumor suppressor functional requirements in multiple tissue lineages and cells. In addition, the latency of the different *Pten*-deficient cancers provides a valuable time frame for a more detailed dissection in the future of the downstream effectors of *Pten* loss using pharmacological or genetic approaches. In this study, we tested the pharmacological inhibition of the *Pten* downstream signaling cascade using the PI3K inhibitor, LY294002, which has been demonstrated to suppress tumor outgrowth in xenografted ovarian tumors and pancreatic cancer cells in the nude mice [37], [38]. Recently, Shukla *et al* showed that LY294002 treatment suppresses the invasive properties of several human prostate cancer cell lines [39]. In our study, we found that prostate hyperplasia after induced *Pten* loss at an adult stage is suppressed by LY294002. To our knowledge, this is the first evidence to show tumor inhibitory activity of LY294002 in spontaneous tumor formation using a genetically engineered mouse model. Moreover, recent reports have shown that mTOR inhibition effectively suppresses neuronal hypertrophy, endometrial hyperplasia and leukemia initiation after tissue-specific *Pten* loss or in the *Pten^+/−^* mice [24], [40], [41]. Likewise, mTOR inhibition could also be tested in our temporal controlled *Pten*-deficient tumor model and this will allow the dissection of mTOR pathway requirements when *Pten* is lost in systemic organs.

In summary, we utilized an inducible Cre/*lox*P system to ablate *Pten* in a temporal-specific manner in the adult mouse. Through the monitoring of tumor spectrum, tumor latency and tumor incidence in the *Pten*-deficient mice, our findings provide an entry point for an understanding the role of *Pten* in tumorigenesis across different tissues. Furthermore, these 4OHT-inducible *R26CreER^T^; Pten^fx/fx^* mice will be a useful mouse model that can be used to identify and study potential therapeutic approaches that will prevent *Pten*-deficient malignancies.

## Materials and Methods

### Mice

*Pten^fx/fx^* [28], [29], *ROSA26 Cre reporter (R26R)* [42] and *R26*-*CreER^T^* [27] mice were obtained from the Jackson Laboratory (Jackson Laboratory, Bar Harbor, ME, USA). *R26-CreER/+;R26R/+* bigenic mice were generated in order to examine the effect of inducible Cre activity in the systemic organs. *R26-CreER^T^/+;Pten^fx/fx^* and the control (*R26-CreER^T^/+;Pten^fx/+^* or *Pten^fx/fx^*) mice were generated in a mixed background (BALB/c;129;C57BL/6) by crossing *Pten^fx/fx^* and *R26-CreER^T^* mice; this was followed by crossing *R26-CreER^T^/+;Pten^fx/+^* and *Pten^fx/fx^* mice. Up to five mice were housed in microisolator cages and provided with autoclaved food and water in a specific pathogen free colony. The experimental procedures using animals were in accordance with the guidelines of Institutional Animal Care and Use Committee. PCR genotyping was performed according to the standard protocols.

### Pharmacologically administration

4-hydroxytamoxifen (4-OHT; Sigma-Aldrich, St. Louis, MO, USA) was dissolved in dimethyl sulfoxide (DMSO; Sigma-Aldrich) at a concentration of 10 mg/ml. 4-OHT solution was emulsified in sunflower seed oil (Sigma-Aldrich) by vortexing, which was followed by mixing on a rotator for 4∼6 hours. The mice at the age of 6 weeks were injected intraperitoneally with a dose of 4-OHT (4∼5g per gram body weight) every other day three times. Similarly, LY294002 (Calbiochem, La Jolla, CA, USA) was prepared using the same procedure as 4-OHT. For the LY294002 administration, mice were injected intraperitoneally with LY294002 (4∼5g per gram body weight) one week after the 4OHT treatment.

### Whole mount X-gal staining

The systemic organs including brain, lung, liver, spleen, pancreas, lung, kidney, stomach, intestine and reproductive system were dissected. Organs were fixed transiently with 10% neutral-buffered formalin for 30 minutes and rinsed with rinse buffer (2 mM MgCl_2_, 0.1% sodium deoxycholate, 0.2% NP-40, 1× PBS, pH7.3) for 30 minutes. Organs were incubated with X-gal staining solution (5 mM potassium hexacyanoferrate, 5 mM potassium hexacynoferrate trihydrate, 1 mg/ml X-gal in rinse buffer) for 1 hour at 37°C or room temperature with gentle agitation until the appearance of blue coloration and then organs in X-gal staining solution were transferred to 4°C refrigerator overnight. Finally, stained organs were post-fixed with neutral-buffered formalin overnight and stored in 70% ethanol at 4°C or photography examined by stereomicroscopy (MZ6, Leica, Germany).

### Analyses of tumor-free survival and tumor malignancy

The tumor-free survival rate was analyzed using the Kalpan-Meier method by the SPSS program (SPSS, Inc., Chicago, IL, USA). Detail analysis of tumor formation has been described previously [43], [44]. Briefly, mice were inspected for illness or tumor formation twice every week. Normal tissues or tumor samples were isolated and fixed in 10% neutral-buffered formalin at 4°C overnight. Subsequently, the samples were embedded in paraffin and further processed for histopathological studies as described below. Lung, kidney, liver and enlarged lymph nodes were also isolated to histologically to be examined for metastasis. If tumors were not identified by gross examination, then a necropsy was performed. Diagnosis of tumor malignancy was carried out in consultation with pathologists. In general, tissue sections stained by hematoxylin and eosin (H&E; see below) were examined by light microscopy (BX51, Olympus, Japan). Tumor malignancy was characterized by invasive behavior or metastasis. Expansive tumor lesions, but with a relatively normal cytological appearance and not showing an invasive behavior, were considered as benign or pre-cancerous.

### Hematoxylin and eosin (H&E) staining

Tissue sections on slides were deparaffinized and rehydrated and this was followed by staining with Harris Hematoxylin (DakoCytomation, Denmark) for 3∼5 minutes. Sections were washed with ddH_2_O and then transferred to Tris-HCl (pH 8.0) for 5 minutes. Subsequently, the sections were stained with Eosin Y (Shandon, Tokyo, Japan) for 30 seconds, dehydrated and then mounted.

### Immunohistochemical (IHC) and immunofluorescence studies (IF)

Tissue sections were deparaffinized, rehydrated and placed in boiling antigen retrieval buffer (DakoCytomation). Endogenous peroxidase activity was quenched by 3% hydrogen peroxide for 5 minutes and this was followed by incubation with blocking reagent (1% Cold Water Fish Skin Gelatin, 5% Bovine Serum Albumin in 1× Phosphate Buffered Saline) for 1 hour at room temperature. For IHC, the tissues sections were subsequently incubated with primary antibodies against CD3 (ready to use; Abcam Ltd, Cambridge, UK), B220 (1∶50 dilution; Southern Biotech, Birmingham, AL, USA;), E-cadherin (1∶50 dilution; DakoCytomation) and p-AKT(Ser473) (1∶100 dilution; Cell Signaling Technology, Beverly, MA, USA) for 1 hour at room temperature The tissue sections was then incubated with biotinylated-linked secondary antibody (anti-mouse/rabbit; DakoCytomation) for 30 minutes and Streptavidin-HRP (DakoCytomation) was added to amplify the signals. Staining was performed using a NovaRed substrate kit (Vector Laboratories, Burlingame, CA). The slides were counterstained for a short time with Hematoxylin, which was followed by dehydration and then mounting. For IF, tissues sections were incubated with primary antibodies against PTEN (1∶12500 dilution; Cascade BioScience, Winchester, MA), cytokeratin 5 (CK5, 1∶400 dilution; Abcam) and/or cytokeratin 8 (CK8 or TROMA-1, 1∶400, Developmental Study Hybridoma Bank, University of Iowa, Iowa City, IA, USA). Secondary antibodies (AlexaFluor 568 conjugated anti-rat IgG against antibody to CK8 and AlexaFluor 488 conjugated anti-rabbit IgG against antibody to CK5; Molecular Probes, Invitrogen) were next incubated on the slides for 1 hour and this was followed by DAPI staining for 2 minutes. A tyramide signaling amplification (TSA) system (kit#22 with HRP-streptavidin and AlexaFluor 488, Invitrogen) was used to enhance the PTEN signal. Finally, the slides were further incubated with DAPI for 2 minutes and mounted in fluorescent mounting medium (DakoCytomation).

### Flow cytometry analysis

The control or enlarged lymphoid organs (thymus and lymph nodes) were dissected and collected in DMEM medium. These lymphoid tissues were grounded and filtrated through cell strainers. The cells present were counted and then centrifuged for 5 minutes at 4°C. Then, the cell suspensions were incubated with 2.4G2 monoclonal antibody (a gift from Dr Jeffery J.Y. Yen) for 30 minutes on ice followed by labeling with FITC-conjugated antibodies against CD4 (eBioscience, San Diego, CA, USA) and PE-conjugated antibody against CD8 (eBioscience) for 15 minutes. After washing the cell pellets twice with 1xPBS, the cells were resuspended in 1xPBS containing Propidium Iodine (1g/ml, eBioscience). The percentage of live cells (PI exclusive) expressing the CD4/CD8 surface markers were analyzed by flow cytometry (FACScalibur, Becton Dickinson, USA) using Cell Quest software.

## Supporting Information

Figure S1 Immunofluorescence analysis of PTEN expression. After one week of 4OHT injection, PTEN expression was determined using antibody against PTEN (green) and counterstained with DAPI (blue) in the lung and the kidney of the *R26-Pten^fx/+^* and *R26-Pten^fx/fx^* mice.
(1.01 MB TIF)

Figure S2 *R26-Pten^fx/+^* malignancies. (A) H&E-stained section of a lung cancer shows the bronchioloalveolar features found in a *R26-Pten^fx/+^* male at 42 weeks post 4OHT treatment. (B) H&E-stained section showing lung cancer metastasis in the liver of the same mouse described in A. (C) H&E-stained section of a mammary gland tumor showing lobular intraductal proliferation and microinvasion patterns in a *R26-Pten^fx/+^* female at 52 weeks. (D) H&E-stained section of a liver cancer showing disarrangement of the sinusoids, trabecular or pseudoglandular features with scattered hyaline bodies in a *R26-Pten^fx/+^* male at 58 weeks. High magnification views in *C* and *D* showing mitotic figures (arrowheads) have been inserted (Bar, 100m). Bars in *A∼D*, 200m. T, tumor lesion.
(1.91 MB TIF)
