# Supplementary figures and images for: Tumor Spectrum, Tumor Latency and Tumor Incidence of the Pten-Deficient Mice
Source: PLoS One. 2007 Nov 28;2(11):e1237. doi: 10.1371/journal.pone.0001237 (PMC2077932; doi:10.1371/journal.pone.0001237)

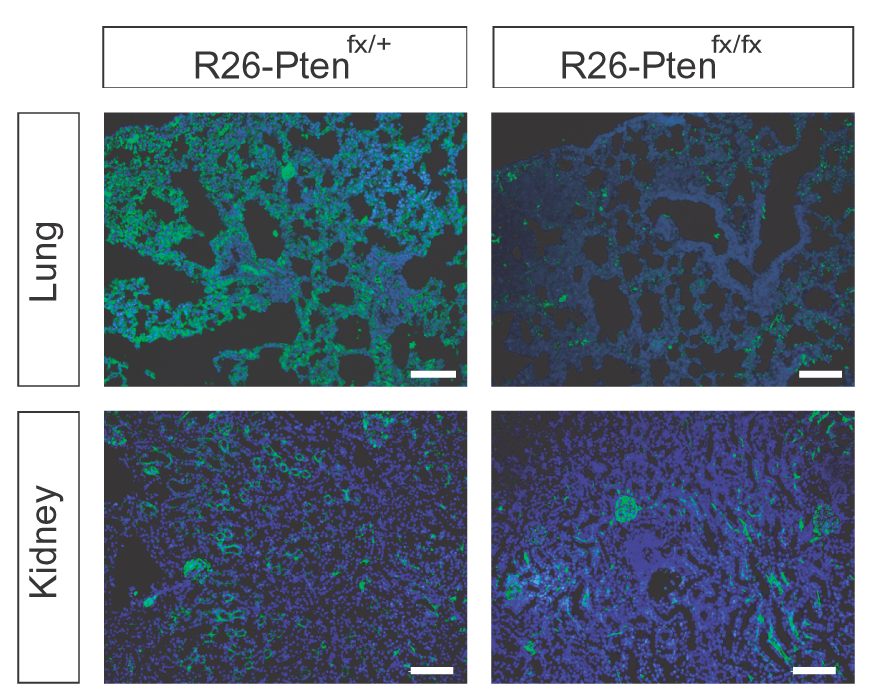

Supplement: Figure S1 — Immunofluorescence analysis of PTEN expression. After one week of 4OHT injection, PTEN expression was determined using antibody against PTEN (green) and counterstained with DAPI (blue) in the lung and the kidney of the R26-Ptenfx/+ and R26-Ptenfx/fx mice. (1.01 MB TIF) [file pone.0001237.s001.tif]

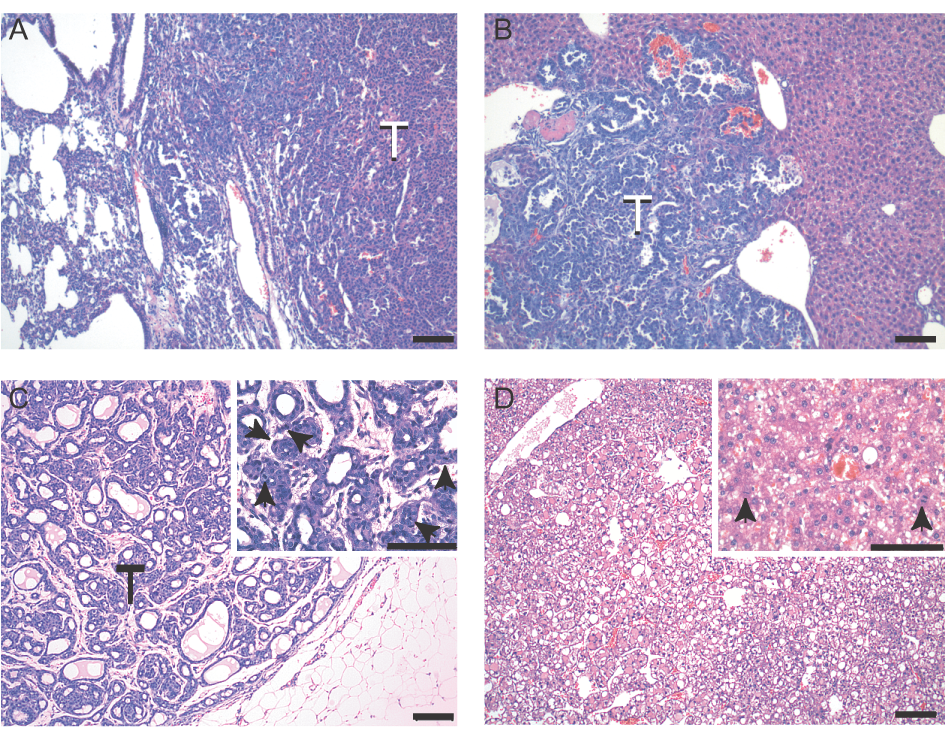

Supplement: Figure S2 — R26-Ptenfx/+ malignancies. (A) H&E-stained section of a lung cancer shows the bronchioloalveolar features found in a R26-Ptenfx/+ male at 42 weeks post 4OHT treatment. (B) H&E-stained section showing lung cancer metastasis in the liver of the same mouse described in A. (C) H&E-stained section of a mammary gland tumor showing lobular intraductal proliferation and microinvasion patterns in a R26-Ptenfx/+ female at 52 weeks. (D) H&E-stained section of a liver cancer showing disarrangement of the sinusoids, trabecular or pseudoglandular features with scattered hyaline bodies in a R26-Ptenfx/+ male at 58 weeks. High magnification views in C and D showing mitotic figures (arrowheads) have been inserted (Bar, 100m). Bars in A∼D, 200m. T, tumor lesion. (1.91 MB TIF) [file pone.0001237.s002.tif]
